# Evaluation of Organic Wastes as Substrates for Rearing *Zophobas morio*, *Tenebrio molitor*, and *Acheta domesticus* Larvae as Alternative Feed Supplements

**DOI:** 10.3390/insects11090604

**Published:** 2020-09-05

**Authors:** Endre Harsányi, Csaba Juhász, Elza Kovács, László Huzsvai, Richárd Pintér, György Fekete, Zsolt István Varga, László Aleksza, Csaba Gyuricza

**Affiliations:** 1 National Agricultural Research and Innovation Center, Institute of Agricultural Engineering, Tessedik Sámuel 4, H-2100 Gödöllő, Hungary; harsanyi.endre@mgi.naik.hu; 2 Faculty of Agricultural and Food Sciences and Environmental Management, University of Debrecen, Böszörményi 138, H-4032 Debrecen, Hungary; ekovacs@agr.unideb.hu; 3 Faculty of Economics and Business, University of Debrecen, Böszörményi 138, H-4032 Debrecen, Hungary; huzsvai.laszlo@econ.unideb.hu; 4 Faculty of Food Science, Szent István University, Villányi 35-43, H-1118 Budapest, Hungary; Richard.Pinter@phd.uni-szie.hu; 5 Faculty of Agricultural and Environmental Sciences, Szent István University, Páter Károly 1, H-2100 Gödöllő, Hungary; Fekete.gyorgy@mkk.szie.hu (G.F.); Varga.Zsolt.Istvan@mkk.szie.hu (Z.I.V.); gyuricza.csaba@mkk.szie.hu (C.G.); 6 ProfiKomp Environmental Technologies Inc., Kühne Ede 7, H-2100 Gödöllő, Hungary; alexa@profikomp.hu

**Keywords:** larva, growth, nutritional value, principal component analysis

## Abstract

**Simple Summary:**

The yellow mealworm, super worm, and house cricket are among the most widely produced insects, with high feed conversion efficiency. However, their nutritional composition and development rate generally vary with environmental conditions. From an economic point of view, rearing conditions such as diet, temperature, and time, and insect performance such as mortality and nutritional value are the most important factors. During their intensive growth period at room temperature, when fed a diet of vegetable waste, garden waste, cattle manure, or horse manure with 10% chicken feed, the worms performed better in terms of survival, but presented lower weight. Moreover, they showed differences in growth intensity with time. The yellow mealworm and super worm presented a relatively high fat concentration. House cricket is specifically high in protein; however, its nutritional composition is affected by the rearing substrate, and it was more sensitive to the low-value substrates. Feeding nutrient-poor diets resulted in a low protein concentration and high fat concentration in the larvae of all three species.

**Abstract:**

Studies have focused on identifying combinations of insects and organic waste to optimise bio-conversion. Here, the effects of different diets (10% chicken feed complemented with 90% vegetable waste, garden waste, cattle manure, or horse manure) on growth and survival rates, and nutritional value of *Zophobas morio* and *Tenebrio molitor* larvae, and *Acheta domesticus* were investigated. Compared with chicken feed, organic waste decreased the individual larval weight, although green waste showed fewer negative effects than the manure. The macronutrient concentrations in garden waste were moderate compared with chicken feed, and vegetable waste was the poorest diet in terms of nutrient concentration, as revealed by the principal component analysis (PCA). There was no difference in weight between larvae reared on garden waste and those reared on vegetable waste. *Tenebrio molitor* and *A. domesticus* showed the maximum growth rates at 71–101 and 36–66 days of age at 22.5 ± 2.5 °C, respectively. The PCA showed that the protein and fibre concentrations were inversely proportional to fat concentration in the larvae. *Acheta domesticus* was rich in proteins, whereas *Z. morio* and *T. molitor* were rich in fat. Feeding nutrient-poor diets resulted in a lower protein and a higher fat concentration in the larvae.

## 1. Introduction

Edible insects are of high nutritional value, and they can be used to partially replace protein ingredients in compound feeds for common livestock and aquaculture. Furthermore, insects of different compositions can be considered an ingredient in feed formulations or can be added to diets [1]. The most common commercial insect species are the yellow mealworm (*Tenebrio molitor* L.), super worm (*Zophobas morio* Fab.), housefly (*Musca domestica* L.), black soldier fly (*Hermetia illucens* L.), house cricket (*Acheta domesticus* L.), and greater wax moth (*Galleria mellonella* L.) [2,3,4,5]. The composition of insect larvae varies with species, and it may be either species-specific or modified by their diet. Oonicx et al. [6] reported that variations in protein and fat concentrations in feed substrates were not reflected in their concentrations in yellow mealworm and house cricket. Argentinian cockroaches (*Blaptica dubia*) reared on a high-protein, low-fat diet contained almost two times more crude proteins than those reared on a low-protein, high-fat diet. The black soldier fly fed a high-protein/high-fat diet showed an elevation in protein concentration, and this suggests that the variation in the nutritional value of insects is species-specific; Shumo et al. [7] also reported the same.

For large-scale production of insect-based feed supplements, the cost of rearing substrates is a key issue. The major expenses in insect rearing are related to procuring raw materials used for feeding the insects [8] and maintaining elevated temperature for most species [9]. In the European Union, edible insects can be fed only materials of vegetable origin and certain materials of animal origin. However, they can grow efficiently on bio-waste and by-products such as restaurant and household waste, slaughterhouse by-products, animal manure, and gardening waste [10]. Thus, feed regulations should be revised to encompass these alternatives in the circular economy; these raw materials should be authorised for utilisation [11]. Studies have focused on rearing insects on low-value substrates, such as organic wastes and by-products, and indicated their advantages and limitations. Nguyen et al. [12] compared the effects of liver, manure, kitchen waste, fruits and vegetables, and fish with those of poultry feed on the performance of *H. illucens* larvae. The larvae that were fed manure were shorter, lighter weight, and took longer to develop, whereas those fed kitchen waste were longer and heavier. Furthermore, Nguyen et al. [13] reported that the mean consumption rate of larvae was the highest for kitchen waste, but the larvae showed variable nutritional concentration. Shumo et al. [7] compared the nutritional value of *H. illucens* larvae reared on chicken manure, brewers’ spent grains, and kitchen waste, and found differences in the concentrations of crude proteins, ether extract, minerals, amino acids, acid detergent fibre, and neutral detergent fibre. Varelas and Langton [14] reviewed the potential of forest biomass by-products for rearing edible insects and provided examples of unbalanced feeding and its negative consequences on growth and nutritional values. Kim et al. [15] investigated the effect of agricultural waste as a feed for *T. molitor* by replacing mushroom substrates with wheat bran feed. Although the larvae thus reared were lighter and required longer development periods than the control group larvae, their survival rate was similar to that of larvae reared on mushroom substrates. A study on the performance of *H. illucens* larvae [16] showed that the total mean yield and average individual weight of the larvae reared on restaurant food waste were the highest, followed by brewer’s waste, faecal sludge, and banana peels. Additionally, the concentration of crude proteins was significantly higher in the larvae reared on restaurant food waste and brewer’s waste.

Zaelor and Kitthawee [17] evaluated the effect of larval density on productivity in *T. molitor* and *Z. morio*, and reported that the mixture of wheat bran and ivy gourd leaves was not an ideal diet for rearing either species. Lundy and Parrella [18] demonstrated that unprocessed and lower-quality organic side-streams, including post-consumer food waste, could not support the adequate growth and survival of *A. domesticus* populations. Jucker et al. [19] assessed the suitability of organic waste from cricket and locust farming for *H. illucens* production, and found that a higher protein concentration resulted in higher feed conversion. However, the final protein and fat concentrations in the prepupae only partially reflected their concentrations in the rearing substrates. Varelas and Langton [14] evaluated the roles of macronutrients and micronutrients (including carbohydrates, lipids, proteins, sterols, vitamins, and minerals) in insect growth and reproduction, and emphasised the importance of nutrition in obtaining larvae with a high performance. Furthermore, they provided examples of unbalanced feeding and its negative consequences. Oonicx et al. [6] fed by-products of the food industry to *T. molitor* and *A. domesticus*, identified diets that can be as efficiently converted by the larvae as pigs, and concluded that when fed optimal diets, larvae can convert their feed as efficiently as poultry, after correcting to the edible portion. Especially for nitrogen efficiency, their performance was higher than that of conventional production animals. Diet affected the survival and development period of *T. molitor*; furthermore, feeding carrots increased the dry matter content and nitrogen efficiency, and decreased the development period. Contrarily, Liu et al. [20] reported that wheat bran supplemented with approximately 30% fresh plant materials did not significantly affect mealworm survival, development, and proximate composition; however, its growth rate and final weight were improved. Lundy and Parrella [18] measured the biomass output and feed conversion ratio of *A. domesticus* reared on diets ranging from grain-based to highly cellulosic diets, and concluded that the nitrogen concentration, N-to-acid detergent fibre concentration ratio, and crude fat concentration explained most of the variabilities among feed treatments. However, the mortality rate of crickets fed minimally processed, municipal-scale food waste and diets composed largely of straw was >99% without reaching a harvestable size. Some studies have revealed that low-value diets may be effective even at temperatures lower than the optimal range [21,22].

On the basis of the above findings, the following can be inferred: (i) the growth of larvae can be maximised by nutrition; (ii) chemical composition can be functionally pre-designed; and (iii) low-value, organic by-products and waste have great potential as substrates for increasing economic feasibility in insect rearing. Larval performance and waste biotransformation depend on the chemical composition of the organic by-products selected. Therefore, the objective of the current study was to compare the effects of two types of green waste and two types of manure as rearing substrate on the larval performance of three edible species, *T. molitor*, *Z. morio,* and *A. domesticus*, under the same environmental conditions, during their intensive growing period. The substrates were evaluated for their effect on growth, nutritional values, and mortality of the larvae.

## 2. Materials and Methods

### 2.1. Insects and Diets

For the study, the superworm (*Zophobas morio*, Coleoptera: Tenebrionidae), yellow mealworm (*Tenebrio molitor*, Coleoptera: Tenebrionidae), and house cricket (*Acheta domesticus*, Orthoptera: Gryllidae) were selected among the seven commercially available insect species for feeding animals in the EU. *Acheta domesticus* and *T. molitor* are among the most intensively investigated species, whereas *Z. morio* has not been extensively studied, although it is also mass-produced. The insects were procured from BUGS-WORLD Ltd. (Tiszakécske, Hungary). The organic wastes were:

1. Vegetable waste (mixed peels of 10% onion, 25% potato, 25% sweet potato, 30% carrot, and 10% cucumber, with a total water content of 91.4%);
2. Green garden waste with grass (50% *Poaceae* species and other common weeds, 25% tree leaves, and 25% branches (*Populus*, *Salix*, *Pinus,* and *Corylus* species), and a mixture of stone fruits, and other ornamental plant parts, with a water content of 36.2%;
3. 55% cattle manure with faeces and urine, and 45% cereal straw with a water content of 45.7%;
4. 35% horse manure with faeces and urine, and 65% cereal straw with a water content of 28.3%.

For the experiments, the larvae were fed diets (henceforth called substrates), containing 90% of the given organic waste and 10% of chicken feed. The green waste was chopped to 2–4-cm long pieces and manures were broken into small pieces. Mashed chicken feed, produced by VITAFORT Plc. (Dabas, Hungary) for intensive broiler breeding (13.05% water, 0.80% lysine, and 0.30% methionine), was mixed with the substrates and was used as a control. Chicken feed was chosen considering both industrial practice and recommendations, and previous study results [23,24,25,26]. Preliminary tests showed a high mortality of insects with all combinations of the selected organic waste in pure form. However, providing a limited amount of balanced feed improved larval survival; 10% chicken feed supplementation generally resulted in a low mortality rate.

### 2.2. Experimental Design

Before starting the feeding experiments, the newly hatched larvae of *T. molitor* and *Z. morio*, and those of *A. domesticus* were reared on chicken feed, fresh carrots, and cucumber (70%, 20%, and 10%, respectively) for 56 and 21 days, respectively, when they started to grow intensively. The diet provided during this period ensured that the larvae were in good condition at the start of the experiment. Air humidity was maintained at 60% ± 4% for *T. molitor* and *Z. morio*, and 80%, 70%, and 60% for *A. domesticus* during the subsequent weeks to provide optimal conditions for healthy insect development. In the rearing environment, the temperature was 22.5 ± 2.5 °C and humidity was 60% ± 4%, with a 12:12-h light/dark cycle. We focused on the intensive growing period of the selected species under the given rearing conditions. The experiments lasted for 45 days with all three insects. The period was determined by considering the relationship between temperature and insect weight, on the basis of the results of Adámková et al. [27] and Rodjaroen et al. [28] for *T. molitor*, Kulma et al. [29] for *Z. morio*, and Morales-Ramos et al. [9] and Booth and Kiddell [22] for *A. domesticus.* The initial number of larvae was 100 in each trial, with three replications. The larvae were fed ad libitum throughout the experimental period. Fresh substrates of weight equivalent to 25× the net weight of live larvae were added, and the residues and excreta were removed on day 15 and 30 when recording the weight of the larvae. The size of the plastic box (width × length × height) for *T. molitor* and *Z. morio* was 30 × 38 × 10 cm, and that for *A. domesticus* was 18.1 × 25.6 × 13.6 cm; egg cartons with a surface area of approximately 1800 cm^2^ were used in each trial.

### 2.3. Measured Parameters

Mortality, growth, and weight of live larvae were recorded on day 15, 30, and 45. After maintaining the larvae at 4 °C for 60 min, they were separated from the remaining substrate and excreta with a spatula in the first period (later by sieving with mesh of size 2 mm), and then further separated as dead and alive. Only live individuals from each experimental unit were considered when recording. The larvae were weighed using a pre-calibrated, KERN ABT 320-4NM analytical balance of 0.1 g weighing accuracy, with a measuring range of 10 mg–320 g. Nutritional composition of the larvae, including crude protein, crude fat, fibre, ash, and energy, was measured after 45 days of the experiment. The substrates were analysed for the total organic content, and total nitrogen, protein, carbohydrate, fat, total phosphorous, potassium, and calcium concentrations. All chemicals and reagents were of analytical grade. Total nitrogen concentration was determined using the Kjeldahl method according to the standard ISO 5983-1:2005 method for animal feedstuff. Crude protein concentration (P) was calculated using Equation (1)

P = total Kjeldahl nitrogen × CF,(1)

where CF is the conversion factor, which is 4.76 for the larvae and 6.25 for the substrates. Janssen et al. [30] proved that nonprotein N in insects leads to an overestimation of protein concentration. They reported comparable CF values among larvae belonging to different orders; the CF for *T. molitor* was 4.76 ± 0.09. Crude fat concentration in the substrates and the larvae was determined using the standard ISO 11085:2015 method for cereals, cereal-based products, and animal feedstuffs using a VELP Scientifica^TM^ SER 148 series semi-automatic extractor. Carbohydrate concentration was determined using a spectrophotometer (Hach DR6000), according to the method of Dubois [31]. Fibre concentration was measured according to the method of Bordereau and Andersen [32] for termite species. Total ash concentration was determined using the standard ISO 936:2000 method for meat and meat products. Gross calorific value was determined using the standard ISO 9831:1998 method with a Parr 6400 automatic isoperibol oxygen bomb calorimeter. Substrate extracts were prepared using the microwave-assisted digestion method with 2 mol/dm^3^ nitric acid and 30% m/m hydrogen peroxide, on a Milestone MLS 1200 Mega high-performance microwave digestion unit. The concentration of total organic carbon (TOC), phosphorous, and potassium was determined using the Hach DR6000 spectrophotometer following the LCK 381, LCK 350, and LCK 228 tests, respectively. Calcium concentration in the extracts was measured using a Jenway PFP7 type low-temperature, single-channel, flame photometer.

### 2.4. Data Analyses

The changes in mortality and individual weight of the larvae over time were analysed using a repeated-measures ANOVA, where the larval characteristics were considered the dependent variables, whereas species and rearing substrates were considered the independent variables. The dependent variables were continuous, whereas the responding ones were categorical. The four timepoints (days 0, 15, 30, and 45) were chosen as the repeated measure factor. Time, species, and substrate interactions were considered statistically different at the significance level of 5%. When the F-test results were significant, Duncan’s new multiple range test was used for post-hoc comparison of the differences between the pairs of means at a level of α = 0.05. The relationships between the variables characterising the nutritional value of the substrates and larvae were analysed using a principal component analysis (PCA). For substrates, there were eight variables and 30 observations (five types of substrate and six replications), whereas for larvae, there were five variables and 45 observations (five types of substrate, three species, and three replications). Sampling adequacy was proved using the Kaiser–Meyer–Olkin test. The appropriate number of components for extracts was determined based on Cattell’s scree plots of the successive eigenvalues. To determine the number of principal components, a parallel analysis was performed. Statistical computing was carried out using R software [33]. Data were analysed using R version 3.6.3 (2020-02-29). Figures were created using the package ggplot2 [34].

## 3. Results

### 3.1. Effect of Substrates on Larval Weight

**Figure 1:**
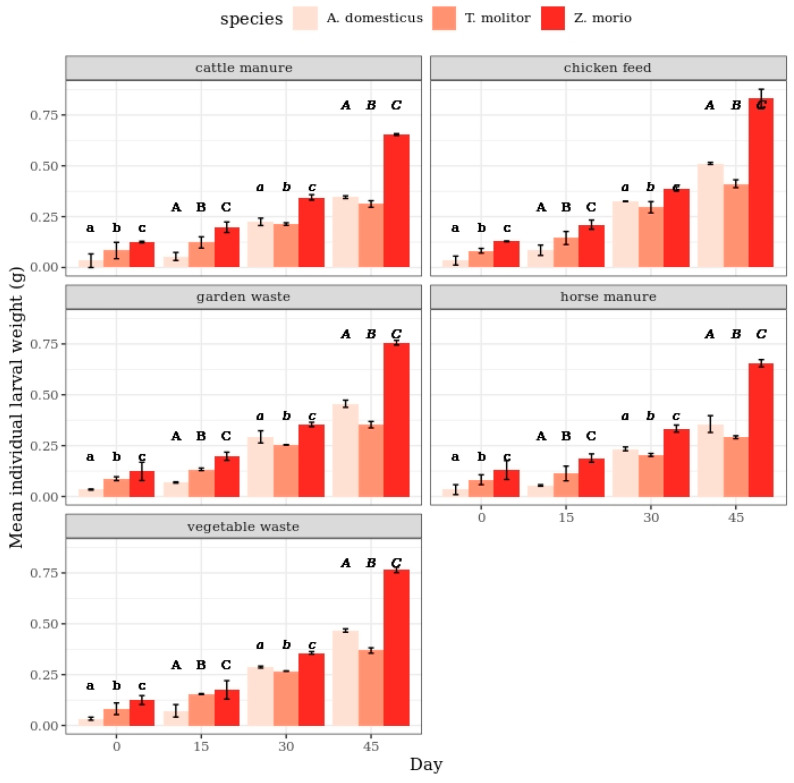
Mean individual larval weight of *T. molitor* and *Z. morio* at the age of 56–101 days, and *A. domesticus* at the age of 21–66 days, by substrates (the bars indicate the 95% confidence interval). Different letters show statistically significant differences among the species.

An increase in the weight of individual larvae was monitored for each species and substrate. There were significant differences between the weights recorded every 15 days for all diets and species (Figure 1). The Duncan test differentiated the three homogenous groups (*p* < 0.10). There were no significant differences in weight among larvae of the three species and between the experimental diets.

**Figure 2:**
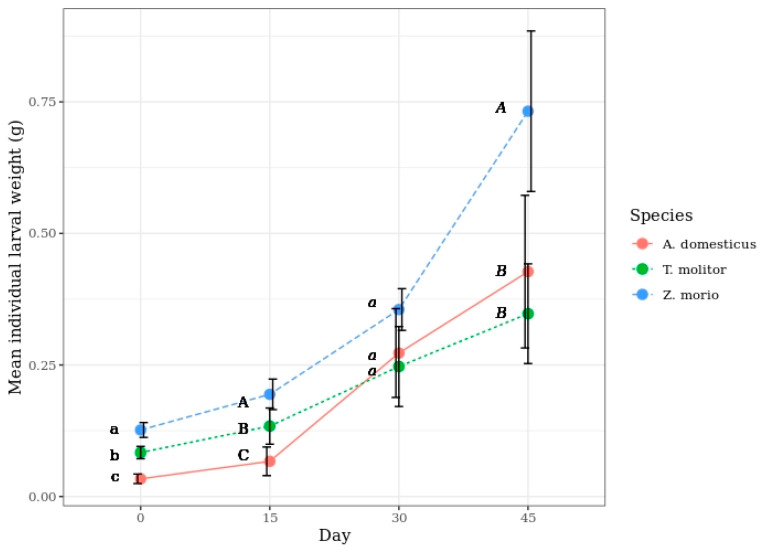
Mean individual larval weight of *T. molitor*, *Z. morio,* and *A. domesticus* when reared on the different substrates, by time (the bars indicate the 95% confidence interval). Different letters show statistically significant differences among the species.

Considering the increase in larval weight during the 45-day experiment period, regardless of the species and rearing substrate, *Z. morio* showed the highest growth rate, followed by *A. domesticus,* and *T. molitor*. The difference in the growth rate between the latter two species changed the order of individual weight between days 15 and 30; it is noteworthy that the initial ages of the larvae were different (21 and 56 days), respectively (Figure 2).

**Figure 3:**
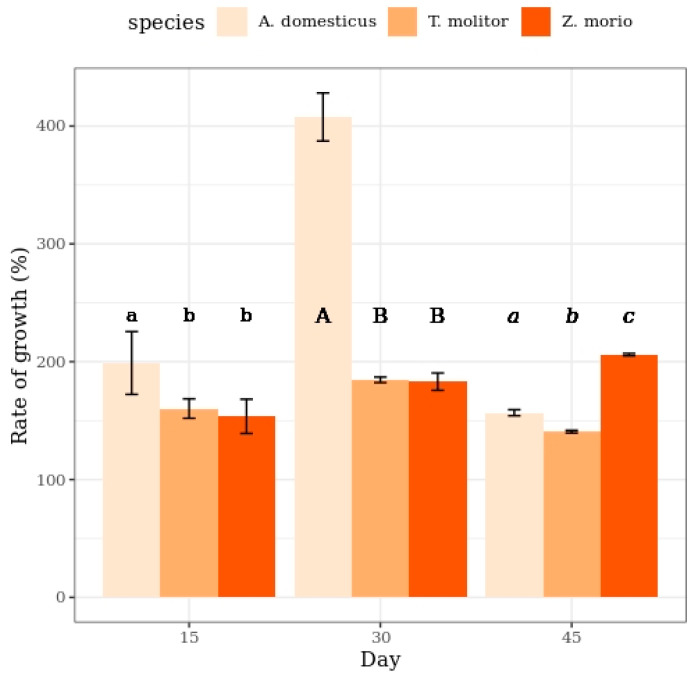
Rate growth of *T. molitor* and *Z. morio* larvae and *A. domesticus* when reared on the different substrates (the bars indicate the 95% confidence interval). Different letters show statistically significant differences among the species.

A comparison between the two species belonging the family Tenebrionidae revealed that *Z. morio* was significantly heavier than *T. molitor* at the age of 56 days (α = 0.05), and this difference increased with time. *Acheta domesticus* larvae were the lightest, but they showed the highest relative increase in individual weight. However, during the experimental period of 45 days, the rate of growth with time, expressed as a rate of change [(m_t_/m_t-1_) × 100], increased for *Z. morio*, started to decrease between days 30 and 45 for *T. molitor*, and peaked between days 15 and 45 for *A. domesticus* (Figure 3).

### 3.2. Nutritional Compositions of the Substrates

The nutrient composition of the substrates used in the experiment is presented in Table 1. The mean dietary ranges for different components were as follows: 9.43–30.08% TOC, 0.16–1.28% N, 27.88–212.80 g/kg protein, 13.35–125.70 g/kg carbohydrate, 2.34–5.58 g/kg fat, 0.10–0.43% K, and 0.79–1.38% Ca. Chicken feed had the highest total nitrogen, protein, and carbohydrate concentrations. The carbohydrate concentration of vegetable waste was comparable with that of other wastes. Fat concentration in the chicken feed was not significantly different from that in vegetable waste and cattle manure, and it was lower than that in garden waste and horse manure. The phosphorous concentration was different in all substrates, whereas the potassium concentration did not differ between the cattle and horse manure.

The PCA revealed that three variables could explain 95.5% of the total variance, and the first two principal components (hereinafter referred to as dimensions) explain 76.6% of the total variance (Figure S1). The first dimension comprised nitrogen, protein, carbohydrate, phosphorous, and potassium, and the second dimension comprised carbon and fat, whereas the third dimension comprised calcium and potassium (Table 2).

**Figure 4:**
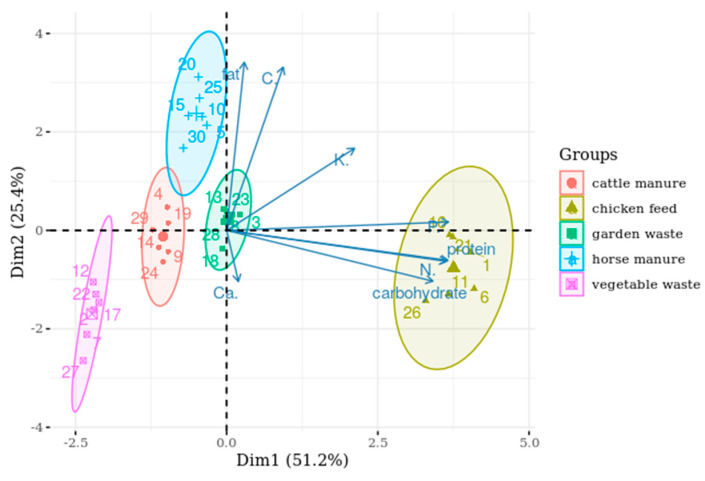
PCA biplot representing the composition of the investigated rearing substrates.

The biplot figures showed that the overall quality of the rearing substrates was comparable in terms of their composition. A vector plot represents variables, where its length shows the level of contribution to a principal component and its direction shows the principal component to which a variable belongs. For optimal visualisation, the x and y axes, representing the first and second components, respectively, were chosen to have non-proportional scaling (Figure 4).

The chicken feed contained the highest concentration of protein, nitrogen, carbohydrate, and phosphorous compared with the other four substrates. The fat and carbon concentrations were relatively higher in horse manure, but they were comparable between cattle manure and garden waste. Vegetable waste had the lowest concentration of fat and carbon.

### 3.3. Nutrient Value of the Larvae

The nutrient composition of the larval species, evaluated at the end of the experiments, showed differences with the diets (Table 3).

The crude protein concentration in *A. domesticus* was 57.8% when fed cattle manure, followed by 56.4% when fed horse manure; the protein concentration was the highest, that is, 67%, when fed chicken feed. The crude fat concentration was 14.4–19.4%, with the lowest and highest concentrations in larvae fed chicken feed and horse manure, respectively. There were no significant differences between the larvae fed manure and those fed garden waste. The fibre concentration ranged from 15.7–19.2%, with the lowest and highest concentrations in larvae fed chicken feed and manures, respectively. The ash concentration was the highest when the larvae were fed garden waste (6.2%), whereas it was the lowest when the larvae were fed the other diets. The larva- fed chicken feed presented the lowest energy level, whereas those fed manure exhibited the highest energy level, with a range of 17.4–18.8 MJ/kg. The crude protein concentration of *T. molitor* was lower, whereas its crude fat concentration was higher than those of *A. domesticus*, with a range of 37.9–47.2% and 43.1–47.5%, respectively. The fibre and ash concentrations were lower in *T. molitor*, whose fibre concentration was half of that of *A. domesticus*. Considering the differences in the nutrient composition of *T. molitor*, rearing the larvae on chicken feed resulted in the highest crude protein and the lowest crude fat concentrations, whereas rearing on horse manure resulted in the highest fat and the lowest protein concentration. Furthermore, the fibre and ash concentrations of the larvae were the lowest when reared on chicken feed, whereas they were the highest when the larvae were reared on the manures. *Zophobas morio* was comparable to *T. molitor* in terms of protein, fat, and ash concentrations. The order of change was similar, with the larvae reared on manure presenting the lowest protein and highest fat concentrations. Only the fibre concentration differed between the diets, wherein garden waste resulted in the highest fibre concentration, whereas horse manure resulted in a low fibre concentration compared with chicken feed.

The Kaiser–Meyer–Olkin test proved that the variables influencing the nutritional value of the larvae were adequate for the PCA, and the measure of sampling adequacy for the individual variables was higher than 0.5. The five main features, namely the protein, fat, fibre, and ash concentrations, and energy level, showed almost a one-dimensional relationship, where one principal component explained 83.8% of the variation (Figure S2). The cumulative sum of the first two principal components explained 97.1% of the variance. The results of PCA for the nutrient composition of larvae are presented in Table 4.

Based on the correlation coefficients, the first dimension comprised crude protein, crude fat, fibre, and energy, and the second dimension comprised ash concentration alone, contributing 13.3% to the overall variation. Evidently, there was a strong positive correlation between crude fat concentration and energy level, whereas the other parameters were inversely proportional, suggesting that the higher the crude fat concentration, the lower the crude protein concentration in the larvae.

**Figure 5:**
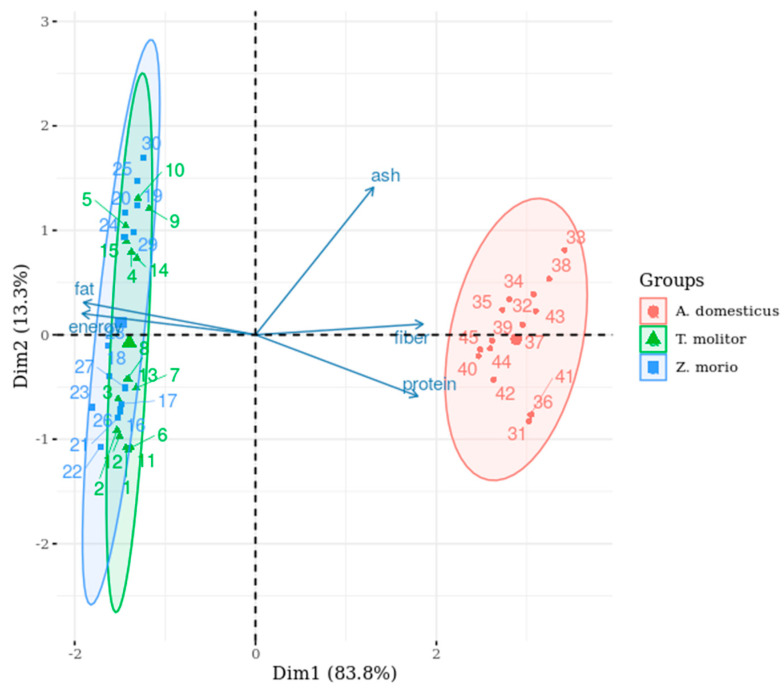
PCA biplot representing the larval macronutrient composition of the investigated species.

For the comparison of the nutrient concentration of the larvae, the PCA biplot revealed two distinct groups (Figure 5). *Acheta domesticus* was rich in proteins and fibre but poor in fat and energy, whereas *T. molitor* and *Z. morio* were rich in fat and energy but relatively poor in protein and fibre, still representing a ratio close to 1:1. The characteristics of the two members of the same insect family showed an overlap.

**Figure 6:**
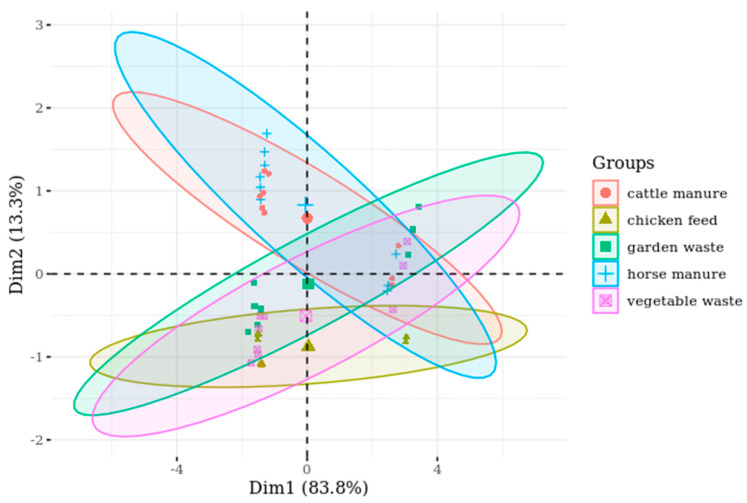
PCA biplot representing larval ash concentration dependency on the rearing substrates.

The mean ash concentration of *A. domesticus* was higher and less variable than that of *T. molitor* and *Z. morio*. The ash concentration of *A. domesticus* was independent of the substrate composition, but manure diet increased and the green waste diet decreased the ash concentration in *T. molitor* and *Z. morio* (Figure 6).

### 3.4. Evaluation of the Effect of Substrates and Rearing Time on the Survival of Larvae

After 45 days of rearing, the highest recorded mortality of *Z. morio* and *T. molitor* larvae was 6.67% ± 0.58% and 2.76% ± 0.58%, respectively. At the end of the experiment, the mortality rate of *A. domesticus* fed chicken feed was 5.30% ± 1.53%. However, after 45 days of rearing, the percentage of live *A. domesticus* larvae was 77.67% ± 1.53% for vegetable waste, 68.00% ± 4.36% for garden waste, 64.33% ± 1.53% for horse manure, and 54.67% ± 1.53% for cattle manure. When fed green garden waste and vegetables, there was a considerable drop in the number of live larvae only on day 45. The results of Duncan’s multiple range test showed significant differences (α = 0.1) in mortality among the larvae reared on chicken feed, garden waste, and manures. The effect of cattle and horse manure on mortality was not different, but that of chicken feed and garden waste was comparable. Although the number of live *A. domesticus* larvae was significantly lower (α = 0.05) than that of the two species belonging to Tenebrionidae, there were no significant differences between *T. molitor* and *Z. morio*. The number of live larvae of all three species significantly decreased with time, and the results of the variance analysis revealed a significant difference in the mortality rate among the species.

The effect of the substrates on the mass of live larvae was significantly different (α = 0.1) among the larvae reared on chicken feed, green wastes, and manures. The effects of vegetable waste and garden waste were similar to those of cattle and horse manure. After 45 days, the mass of live *A. domesticus* larvae was not significantly different from that of *T. molitor* when fed vegetable and garden waste, but it was significantly lower than that of *T. molitor* when fed the manure substrates. The net weight of *A. domesticus* significantly increased between days 30 and 45 only in larvae fed chicken feed and vegetable waste. On day 45, the minimum weight of live *A. domesticus* larvae reared on cattle manure was 18.92 ± 0.60 g, representing 39.0% of larvae fed chicken feed (48.47 ± 0.77 g). The maximum mass of live larvae was produced by *Z. morio* reared on chicken feed (81.44 ± 1.69 g); however, with only 2.76% mortality, this species produced live larval mass of 63.65 ± 0.39 g and 63.75 ± 0.77 g when reared on cattle and horse manure, respectively.

## 4. Discussion

### 4.1. Changes in the Larval Weight in Response to Diet

Patton [35] suggested that substrates containing 30% protein, 37% carbohydrate, and 5% fat as optimal for *A. domesticus*. In this study, the ratio of protein and carbohydrate was opposite in all diets, and even chicken feed (as the control) contained 21% protein, 12.6% carbohydrate, and 3.22% fat. Furthermore, water content has a significant effect. For example, McCluney and Rishabh [36] reported an increase in dry mass of 59% when *A. domesticus* was provided water in addition to a commercially available rodent diet. Here, feeding chicken feed significantly increased the weight of *A. domesticus*, *T. molitor,* and *Z. morio* larvae during the experimental period (α = 0.1), suggesting that the organic waste, particularly mixed vegetable waste, garden waste with green biomass of several species, or cattle manure and horse manure, at high percentages may not be optimal rearing substrates.

The growth characteristics of the insects seem to depend on the substrate. *Zophobas morio* (known as superworms when retained in the larval stage for a longer time) can reach a full size of 50–60 mm. In the study of Mancini et al. [37], the larvae did not reach the pupal stage after 1 year when reared on a low-protein diet; however, Li et al. [38] found that *T. molitor* pupated on day 110 even when reared on wheat-derived feed. Kim et al. [14] reported an increase in the larval period from 77 days to 125 days when reared on a nutrient-poor diet. It must be noted that superworms pupate and complete their metamorphosis when isolated and undisturbed, independent of substrate quality [39]. In addition, they found that when reared on wheat bran and fermented feed under environmental stress, the biomass of larvae of the same age (110 days) was 40% of the weight of those reared without an external stress. Rodjaroen et al. [27] found that the length of *T. molitor* larvae reared on wheat bran and banana became almost constant by 90 days. In the present study, the most intensive growth rate was found at 71–101 days of age. Furthermore, *T. molitor* at 101 days of age presented a maximum mean weight of 0.412 g, whereas 90% horse manure in the diet (with the worst performance) resulted in a 29.4% decrease in its weight. This weight is considerably higher than the results reported by Kim [40], where the larvae reared on wheat bran with additional brewer’s spent grain at 25 ± 3 °C weighed 0.145 g on 102 days and 0.28 g before pupation. Broekhoven et al. [41] also recorded a low average larval weight, where *T. molitor* weighed a maximum of 0.14 g when reared on a diet with a high protein concentration. Mancini et al. [37] reported the final larval weight of 0.168 g when the larvae were reared on brewer’s spent grain and cookies in a 1:1 ratio, with 13.44% crude protein and 11.26% carbohydrate (calculated with CF = 4.76). Kim et al. [40] recorded larval weights of 0.12 ± 0.03 and 0.36 ± 0.06 g at 25 and 30 °C, respectively, suggesting that this species is sensitive to temperature. In this study, the growth rate and survival of *T. molitor* fed low-value diets were comparable with the findings of Adámková et al. [27] at higher temperatures in the last and penultimate instar stages. Zaelor and Kitthawee [16] reported *T. molitor* larval weight of 0.40 ± 0.01 g. This increased up to 0.77 ± 0.23 g at low rearing densities, with considerably higher final individual weight of 1.75 g. In the case of *Z. morio*, Kulma et al. [28] reported that the larvae reached the maximum weight in 90 days at 25  ± 2 °C. In this study, contrary to the findings in *T. molitor*, the growth rate of *Z. morio* continued to slightly increase after the age of 101 days. The maximum weight of *Z. morio* reported by Broekhoven et al. [41] was comparable with that of larvae fed chicken feed diet (0.831 g) at the end of the present study. In their study, the larvae fed a low-protein and starch diet presented a minimum larval weight of 0.42 g, which was lower than the worst-performing manure substrate in the present study (0.65 g). In the case of *A. domesticus*, Morales-Ramos et al. [29] observed peak individual weekly biomass gain on day 56 at 27 °C. In the present study, the larvae presented the highest weight gain at the age of 36–66 days. Compared with the findings of Collavo et al. [42], with a rearing period of 81 days after hatching, the larvae in this study should have reached their final weight. At the age of 66 days, the highest mean weight of *A. domesticus* reared on chicken feed was 0.512 g, whereas this decreased to 0.346 and 0.356 g, respectively, when reared on 90% cattle and horse manure. The increase in weight over time in response to chicken feed diet was comparable to the findings of Lundy and Parrella [17] at the same larval age. Collavo et al. [42] recorded a mean weight of 0.452 g for *A. domesticus* at the same age when reared on a human refuse diet, whereas Lundy and Parrella [17] recorded considerably lower values in response to food waste diets. Vaga et al. [43] reported up to an 80% difference in the weight of *A. domesticus* fed a control diet containing 19.2% crude protein and 37.6% starch, and a red-clover-based diet. Collavo et al. [42] found that an aromatic-arboreal diet is not optimal for *A. domesticus*, and its unbalanced nutritional value promoted cannibalism. As an extreme case, Quek et al. [44] found that *A. domesticus* reared on *Brassica rapa,* mixed either with soybean residue or dog food, did not grow and survive. Overall, a high percentage of mixed vegetable waste, garden waste with green biomass of several species, or cattle manure and horse manure cannot be considered an optimal rearing substrate to grow *A. domesticus*, *T. molitor,* and *Z. morio* larvae.

### 4.2. Nutritional Value of the Larvae

With the hypothesis that diet has a significant effect on the macronutrient composition of larvae, the protein or fat concentrations in diet for a given species can be tailored. It is necessary to test diets that represent a wide range in nutrient concentrations. In this study, chicken feed had the highest protein and carbohydrate concentrations, whereas vegetable waste had the lowest protein concentration with a low carbohydrate concentration, with a difference in means of 86.9% and 84.7%, respectively. The carbohydrate concentration in the waste was not considerably different, whereas the fat concentration in the horse manure diet was 2.38 times higher than that in the vegetable waste. When ranking the rearing substrates based on their nutrient concentrations, the PCA revealed garden waste as average, chicken feed as the best, and vegetable waste as the poorest feed for larvae. Based on the PCA results, the nutritional composition of the larvae appears to be species specific and consistent, despite the large variation in the substrates. *Acheta domesticus* was rich in proteins and fibre, and poor in fat, whereas *T. molitor* and *Z. morio* were rich in fat and relatively poor in proteins. The different species showed significantly different nutritional composition when reared on different diets, and all low-nutrient value substrates resulted in reduced protein concentration and increased fat concentration in all three species.

For *T. molitor* and *Z. morio*, Broekhoven et al. [41] found that the larval protein concentration was relatively stable in diets that differed 2–3-fold in protein concentration and that dietary fat has a significant effect on larval fat concentration. In the present study, the variation in the protein and fat concentrations was low despite the considerable differences in the dietary compositions. Furthermore, the differences between the protein and fat concentrations were lower than the findings of Adámková et al. [27] in the last and penultimate instar stages for both species. They found the protein concentration in *T. molitor* larvae purchased from insect farms (assuming optimal rearing conditions) was 52% (calculated with a CF of 6.25), with a low fat concentration of 31%, whereas the concentration of protein and fat in *Z. morio* was 46% and 35%, respectively. In case of *T. molitor*, González et al. [45] also reported 48.8% protein and 30.7% fat concentrations. Similarly, Ravzanaadii et al. [46] reported a crude protein concentration of 46.4% and crude fat concentration of 32.7% when the larvae were reared on wheat bran and vegetables, whereas Busler [47] reported a higher protein concentration (53.8%), and lower fat concentration (20.0%). Adámková et al. [21] conducted experiments at 17, 23, and 28 °C, and observed the maximum fat concentration at 23 °C in the last and penultimate instar stages. According to Kulma el al. [28], *Z. morio* larvae, reared on a substrate composed of wheat bran and mashed carrots at 25 ± 2 °C, did not show variation in its nutrient composition and protein quality at the age of 60–120 days and reached maximum weight at 90 days. In *Z. morio* larvae reared on wheat, corn, soybean meal, water, fruits, and vegetables, Araujo et al. [48] reported 46.8% protein and 43.3% lipid concentrations, which are similar to the concentrations recorded in larvae reared on chicken feed in this study. In the case of *A. domesticus* lavae, Rumpold and Schlüter [49] reported 71% protein and 18% fat concentrations, which were obtained from a commercial supplier. This is the closest to our findings when the larvae were reared on chicken feed. Despite the wide range in macronutrient concentrations in the substrates, the nutritional composition of *A. domesticus*, *T. molitor,* and *Z. morio* was species-specific. Overall, to produce larvae with a relatively high fat concentration, *T. molitor* and *Z. morio* are good candidates, whereas *A. domesticus* is predominantly a protein source. However, in agreement with the findings of Oonincx et al. [6], by choosing an appropriate rearing material, composition can, to a certain extent, be tailored.

### 4.3. Mass of Live Larvae during Rearing

When Miech et al. [22] tested the performance of Cambodian field crickets (*Teleogryllus testaceus*), they found that the larval survival rates on chicken feed, cereals, and green biomass of different plants were similar to those on mono diets, except that certain weeds resulted in lower weights. Veenenbos and Oonincx [50] did not find any advantage in feeding additional carrots to improve the survival of *A. domesticus*. A 40% decrease in the survival rate of *A. domesticus* just before adulthood was recorded by Vaga et al. [44] in a control substrate. In this study, the survival of *A. domesticus* larvae was influenced by the diet. The survival rate was similar to the findings of Sorjonen et al. [51] for insects reared on alternative diets at the age of 15–45 days, and similarly, low-protein alternative diets resulted in higher mortality. Contrary to our findings, Collavo et al. [42] recorded an almost linear decrease in survival with time at the age of 1–81 days, with a survival rate of 47.5% on human refuse waste, as an example. The effect of temperature lower than the optimal range was presumably low, based on the findings of Lachenicht et al. [52]. Oonincx et al. [6] found that the survival of *T. molitor* on low-protein diets (12.9–14.4%), compared with that on high-protein diets (21.9–22.9%), was low. In this study, *T. molitor* and *Z. morio* did not show a considerable increase in mortality when reared on low-protein diets. The growth rate, biomass accumulation, and population viability were strongly determined by food substrate composition in *A. domesticus*, and the N concentration explained 68% of the variation across treatments, whereas the ratio of N-to-acid detergent fibre explained another 28% of the overall treatment variability [17]. The mass of *Z. morio* and *A. domesticus* increased more than that of *T. molitor*, although the weight of the same number of *A. domesticus* larvae was significantly lower than that of *T. molitor* on day 1 of the experiment. The final net mass was still comparable despite the increase in mortality of *A. domesticus* over time. The mass of live larvae is determined by the mortality and the weight of the individual larvae, and their ratio gives the absolute order of the candidates. In this study, *Z. morio* reared on low-value waste showed the highest performance.

## 5. Conclusions

Our results confirmed that a high percentage of mixed vegetable waste, garden waste with green biomass of several species, or cattle manure and horse manure cannot be considered an optimal rearing substrate to grow *A. domesticus*, *T. molitor,* and *Z. morio* larvae. All low-nutrient value substrates decreased the protein concentration and increased the fat concentration in all three species. Despite the wide range of macronutrient concentrations in the substrates, the nutritional composition of *A. domesticus*, *T. molitor,* and *Z. morio* was species-specific, although the nutritional value of the larvae from the three species was significantly affected by the composition of the rearing substrate. We identified protein and fat combinations for the three species that can be suitable for the feeding programs of animals. In the future, we will evaluate means to prolong the survivability of the larvae at 45 days, and relate this to the possible frequency of substrate provision.

## Figures and Tables

**Table 1:** Nutrient composition of the substrates used as diets for rearing *Zophobas morio*, *Tenebrio molitor,* and *Acheta domesticus* larvae.

Components	CF	CF/VW 1:9	CF/GW 1:9	CF/CM 1:9	CF/HM 1:9
Total organic carbon (C%)	19.74 ± 3.38 ^b^	9.43 ± 1.71 ^d^	16.63 ± 2.25 ^c^	19.66 ± 1.70 ^b^	30.08 ± 2.16 ^a^
Total nitrogen (N%)	1.28 ± 0.12 ^a^	0.16 ± 0.01 ^d^	0.60 ± 0.05 ^b^	0.37 ± 0.03 ^c^	0.30 ± 0.03 ^c^
Protein (g/kg)	212.8 ± 8.31 ^a^	27.88 ± 4.78 ^e^	76.18 ± 7.74 ^b^	39.65 ± 7.11 ^d^	50.33 ± 5.84 ^e^
Carbohydrate (g/kg)	125.7 ± 1.74 ^a^	19.25 ± 0.70 ^b^	14.01 ± 1.03 ^d^	13.35 ± 0.71 ^d^	16.90 ± 0.93 ^c^
Fat (g/kg)	3.22 ± 1.08 ^c^	2.34 ± 0.99 ^c^	4.24 ± 0.70 ^b^	3.19 ± 0.64 ^c^	5.58 ± 0.60 ^a^
Phosphorous (P%)	0.43 ± 0.02 ^a^	0.10 ± 0.01 ^e^	0.18 ± 0.02 ^c^	0.16 ± 0.01 ^d^	0.21 ± 0.02 ^b^
Potassium (K%)	0.49 ± 0.04 ^b^	0.10 ± 0.05 ^d^	0.64 ± 0.06 ^a^	0.37 ± 0.04 ^c^	0.42 ± 0.05 ^c^
Calcium (Ca%)	1.02 ± 0.02 ^c^	0.97 ± 0.07 ^c^	1.38 ± 0.03 ^a^	1.15 ± 0.06 ^b^	0.79 ± 0.03 ^d^

Abbreviations: CF: chicken feed (control), VW: vegetable waste, GW: garden waste, CM: cattle manure, HM: horse manure; 1:9 is the ratio of CF and the given organic waste. The values are presented as mean ± SD in dry weight %, *n* = 6. Means within a row with the same letter are not significantly different.

**Table 2:** Results of the principal component analysis and correlation coefficients by variables and dimensions, characterising the compositions of the rearing substrates.

Components	First Dimension	Second Dimension	Third Dimension
Total organic carbon (C%)	0.250	**0.888**	−0.210
Total nitrogen (N%)	**0.973**	−0.169	0.088
Protein (g/kg)	**0.980**	−0.163	−0.057
Carbohydrate (g/kg)	**0.913**	−0.277	−0.283
Fat (g/kg)	0.078	**0.915**	0.097
Phosphorous (P%)	**0.982**	0.045	−0.154
Potassium (K%)	**0.567**	0.447	**0.662**
Calcium (Ca%)	0.052	−0.278	**0.948**

The bold numbers indicate the components with values higher than 0.5 in the same dimension.

**Table 3:** Analysed nutrient composition of *Zophobas morio*, *Tenebrio molitor*, and *Acheta domesticus* larvae by diet (the values are presented as mean ± SD in dry weight %, *n* = 3).

Components	CF	CF/VW 1:9	CF/GW 1:9	CF/CM 1:9	CF/HM 1:9
*A. domesticus*					
Crude protein (g/kg)	67.25 ± 0.10 ^a^	61.20 ± 0.57 ^c^	65.30 ± 0.50 ^b^	57.80 ± 0.10 ^d^	56.40 ± 0.40 ^e^
Crude fat (g/kg)	14.41 ± 0.03 ^c^	17.10 ± 0.65 ^b^	19.30 ± 0.53 ^a^	18.60 ± 0.29 ^a^	19.40 ± 0.43 ^a^
Fibre (g/kg)	15.72 ± 0.03 ^c^	17.50 ± 0.66 ^b^	17.83 ± 0.38 ^b^	18.60 ± 0.29 ^a^	19.20 ± 0.37 ^a^
Ash (g/kg)	4.80 ± 0.04 ^b^	5.40 ± 0.54 ^b^	6.20 ± 0.39 ^a^	5.20 ± 0.32 ^b^	4.98 ± 0.32 ^b^
Energy (MJ/kg)	17.35 ± 0.10 ^e^	18.22 ± 0.01 ^d^	18.27 ± 0.02 ^c^	18.64 ± 0.01 ^b^	18.78 ± 0.02 ^a^
*T. molitor*					
Crude protein (g/kg)	47.18 ± 0.04 ^a^	46.30 ± 0.47 ^b^	42.30 ± 0.27 ^c^	38.92 ± 0.48 ^d^	37.90 ± 0.29 ^e^
Crude fat (g/kg)	43.08 ± 0.05 ^e^	43.30 ± 0.24 ^d^	45.20 ± 0.43 ^c^	46.70 ± 0.31 ^b^	47.50 ± 0.36 ^a^
Fibre (g/kg)	7.44 ± 0.02 ^d^	8.01 ± 0.37 ^c^	8.90 ± 0.41 ^b^	9.50 ± 0.23 ^ab^	9.30 ± 0.16 ^a^
Ash (g/kg)	3.08 ± 0.05 ^b^	3.01 ± 0.39 ^b^	3.20 ± 0.38 ^b^	4.85 ± 0.21 ^a^	5.30 ± 0.33 ^a^
Energy (MJ/kg)	24.17 ± 0.01 ^d^	24.39 ± 0.02 ^c^	24.59 ± 0.01 ^b^	24.68 ± 0.01 ^a^	24.60 ± 0.02 ^b^
*Z. morio*					
Crude protein (g/kg)	46.79 ± 1.03 ^a^	45.70 ± 0.48 ^b^	41.20 ± 0.31 ^c^	39.40 ± 0.34 ^d^	38.70 ± 0.39 ^d^
Crude fat (g/kg)	42.04 ± 0.74 ^d^	43.20 ± 0.31 ^c^	44.30 ± 0.37 ^b^	45.70 ± 0.41 ^a^	46.30 ± 0.42 ^a^
Fibre (g/kg)	9.26 ± 0.04 ^c^	9.43 ± 0.30 ^c^	11.30 ± 0.11 ^a^	10.20 ± 0.48 ^b^	9.32 ± 0.20 ^c^
Ash (g/kg)	2.61 ± 0.03 ^b^	2.89 ± 0.31 ^b^	3.01 ± 0.15 ^b^	4.70 ± 0.34 ^a^	4.89 ± 0.27 ^a^
Energy (MJ/kg)	24.10 ± 0.02 ^d^	24.43 ± 0.01 ^b^	24.38 ± 0.01 ^c^	24.75 ± 0.01 ^a^	24.75 ± 0.01 ^a^

Abbreviations: CF: chicken feed (control), VW: vegetable waste, GW: garden waste, CM: cattle manure, HM: horse manure; 1:9 is the ratio of CF and the given organic waste. Means within a row with the same letter are not significantly different.

**Table 4:** Results of the principal component analysis, and correlation coefficients by variables and dimensions, characterising the nutrient concentration of the larvae.

Components	First Dimension	Second Dimension
Crude protein (g/kg)	**0.929**	−0.304
Crude fat (g/kg)	**−0.985**	0.160
Fibre (g/kg)	**0.959**	0.053
Ash (g/kg)	**0.675**	**0.730**
Energy (MJ/100 g)	**−0.992**	0.105

Bold letters indicate the components with values higher than 0.5 included in the same dimension.

## Supplementary Materials

The following are available online at https://www.mdpi.com/2075-4450/11/9/604/s1, Figure S1: Contribution of variables explaining the total variance in the substrates composition, based on the PCA, Figure S2: Contribution of variables explaining the total variance in the larvae composition, based on the PCA.
