# Supplementary material for: Evaluation of Organic Wastes as Substrates for Rearing Zophobas morio, Tenebrio molitor, and Acheta domesticus Larvae as Alternative Feed Supplements
Source: Insects. 2020 Sep 5;11(9):604. doi: 10.3390/insects11090604 (PMC7564407; doi:10.3390/insects11090604)
Supplement: Supplementary file 1 [file insects-11-00604-s001.pdf]

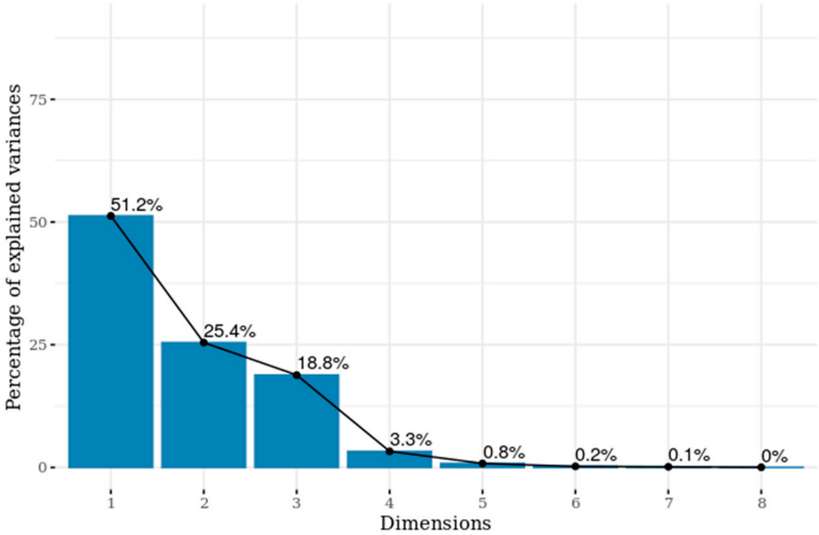

Figure S1. Contribution of variables explaining the total variance in the substrates composition, based on the PCA

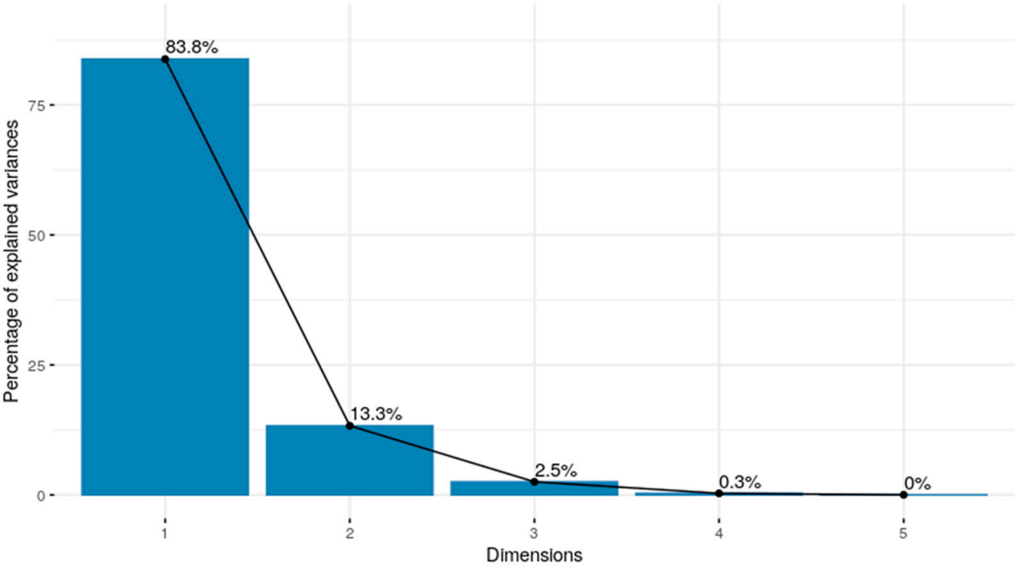

Figure S2. Contribution of variables explaining the total variance in the larvae composition, based on the PCA
